# Piezoelectric Actuators for Compliant Mechanisms

**DOI:** 10.3390/ma19143116

**Published:** 2026-07-20

**Authors:** Simona Noveanu, Dan Cristian Noveanu, Ioan Alexandru Ivan

**Affiliations:** 1 Mechatronics and Machine Dynamics Department, Faculty of Automotive, Mechatronics and Mechanical Engineering, Technical University of Cluj-Napoca, 103 Muncii Avenue, 400641 Cluj-Napoca, Romania; simona.noveanu@mdm.utcluj.ro; 2 European University of Technology, EUT+, European Union; 3 Material Science and Engineering Department, Faculty of Materials and Environmental Engineering, Technical University of Cluj-Napoca, 103 Muncii Avenue, 400641 Cluj-Napoca, Romania; 4 Ecole Centrale Lyon–ENISE, 58 Rue Jean Parot, 42100 Saint-Étienne, France; alex.ivan@infim.ro; 5 Laboratory of Magnetism and Superconductivity, National Institute of Materials Physics, 405A Atomiștilor Street, 077125 Magurele, Romania

**Keywords:** unconventional actuators, piezoelectric actuator, compliant mechanisms

## Abstract

**Highlights:**

**What are the main findings?**

- Stack actuators deliver high force (1000 N) while bending units amplify displacement.
- Parametric simulation matrix integrates material properties with actuator geometry.
- Experimental tests validated the force–displacement models for compliant grippers.

**What are the implications of the main findings?**

- Findings guide the selection of actuators based on specific force-stroke requirements.
- Results optimize the integration of piezoelectric actuators into compliant mechanisms.

**Abstract:**

The growing demand for precision and miniaturization in micro-scale systems and biomedical applications has driven the need for highly optimized compliant mechanisms. The design of these mechatronic systems requires advanced actuators optimization, synchronizing material characteristics, geometric limitations, and mechanical structure. This study presents analysis results, emphasizing the performance of piezoelectric actuators for compliant mechanisms. Analytical results were presented for two types of actuators: stack actuators and bending actuators. We examined displacement when a different voltage was applied and designed a mini gripper as an application. Finally, experimental results for stack and bending piezoelectric actuators are presented. This study provides a solid framework for integrating simulation findings with experimental requirements in the compliant mechanisms field.

## 1. Introduction

Compliant mechanisms achieve motion through elastic deformation of flexible elements rather than through traditional rigid-body joints. These mechanisms offer advantages such as reduced friction, absence of backlash, compact structure, and high precision, making them particularly suitable for micro-scale systems and biomedical applications. However, their performance strongly depends on the selection of appropriate actuation methods capable of delivering precise and controlled deformation while preserving compliance [1,2].

Conventional actuators, such as electromagnetic motors and hydraulic systems, often require rigid transmission components that reduce system compliance and increase complexity. In contrast, unconventional actuators—based on smart materials, magnetic fields, fluidic pressure, or chemical stimuli—can directly induce deformation in compliant structures, improving system integration, efficiency, and miniaturization [3]. Actuators integrated with compliant mechanisms must satisfy several key requirements: compatibility with elastic deformation, high force-to-size ratio, precise displacement control, low mechanical impedance, compatibility with micro-scale fabrication (for MEMS and biomedical systems) and minimal heat generation in biological applications. Unconventional actuators are particularly suitable because they can provide distributed actuation and eliminate the need for rigid transmission elements.

Unconventional actuators can be systematically classified according to the form of energy input and the physical principle used to generate mechanical output. Unlike conventional electromagnetic or hydraulic actuators, unconventional actuators rely on smart materials and field-responsive mechanisms that enable direct energy conversion at multiple scales, particularly in microelectromechanical systems and compliant robotic applications. One major class consists of electrically driven unconventional actuators, which convert electrical energy directly into mechanical deformation through electro-mechanical coupling effects. Piezoelectric actuators operate based on the inverse piezoelectric effect, where the application of an electric field induces mechanical strain in the material, a phenomenon detailed comprehensively in fundamental literature [4] and essential for MEMS thin-film applications [5]. Electroactive polymers (EAP), including dielectric elastomers and ionic polymer–metal composites, generate deformation through electrostatic forces and are capable of high-speed actuation with large strains [6,7]. These actuators are characterized by high precision, fast response time, and excellent scalability, making them particularly suitable for micro positioning and compliant mechanism integration. Recent research has optimized these actuators for specific compliant applications through advanced control strategies [8]. Furthermore, addressing the dynamic challenges of flexible structures under various modal excitations is critical; recent advancements include time-delay measurements and decoupling compensator control designs to improve the stability and performance of flexible beams [9]. For instance, the dynamic characteristics of mass-attached piezoelectric stack actuators have been analyzed to improve performance under load [10], while piezoelectric stick-slip actuators utilizing flexure beams have been developed for enhanced motion ranges [11]. Innovations have also extended to rotary applications [12], precision sensing duties [13], and material processing comparisons [14]. Further studies have explored multistage amplifying mechanisms [15] and flexible tactile sensing attributes [16].

Another important category includes thermally driven actuators, which generate motion through temperature-induced material transformations or differential thermal expansion. Shape memory alloys (SMAs) represent the most widely used thermally activated actuators and operate through reversible phase transformation between martensitic and austenitic crystal structures [17]. This phase transition enables large recoverable strains and high force density, although recent reviews highlight the need for careful design in aerospace and complex loading scenarios [18,19]. Other thermally driven actuators include bimetallic elements and phase-change materials that produce deformation due to thermal expansion differences [20]. These actuators offer compact size and high actuation force but may exhibit slower response due to thermal inertia.

Magnetically driven unconventional actuators represent another important class, in which mechanical motion is generated through magnetic field interactions. These actuators include magneto-active elastomers, magnetic composites, and magnetically controlled micro robotic systems. Magnetic actuation enables wireless control, making it particularly advantageous for biomedical and enclosed environments [21]. Advanced fabrication techniques, such as printing ferromagnetic domains, have further enabled fast-transforming soft materials compatible with compliant mechanisms because they can induce deformation without requiring physical contact or rigid mechanical transmission [22].

Fluid-driven unconventional actuators constitute another major group and operate using pressurized gases or liquids to produce deformation. Pneumatic artificial muscles and fluidic elastomer actuators are typical examples that generate motion through internal pressure changes, a field extensively reviewed for soft robotics applications [23]. These actuators exhibit high compliance and large deformation capability, making them particularly suitable for soft grippers [24] and biological manipulation systems [25]. Hydraulic actuation may also be used in compliant structures when higher force output is required.

Chemically driven unconventional actuators generate motion through chemical reactions, swelling, or other chemically induced structural changes. Stimuli-responsive hydrogels represent a common example and can deform in response to environmental parameters such as pH, temperature, or chemical concentration, often utilized in 4D printing applications [26]. Biohybrid actuators, which utilize living muscle cells, also fall within this category and represent an emerging field of biologically integrated actuation systems.

Optically driven unconventional actuators form another important class and operate by converting light energy into mechanical deformation [27]. Liquid crystal elastomers and photothermal materials deform when exposed to specific wavelengths of light due to molecular reorientation or thermal expansion [28]. Optical actuation enables remote and highly localized control, making it particularly attractive for micro-scale and biomedical applications.

This classification demonstrates that unconventional actuators provide diverse energy conversion mechanisms. However, practical integration requires addressing specific non-linearities. The necessity of hysteresis modeling in piezoelectric actuators has been highlighted [29], and various approaches have been proposed, including Force-Dependent Prandtl–Ishlinskii (FPI) models [30] and Bouc–Wen compensation methods [31]. Furthermore, understanding load-bearing capacity under multi-field coupling [32] and analyzing high-response systems for modular designs is critical [33]. In this paper, we present a comparative analysis of piezoelectric actuators for compliant mechanisms, emphasizing the trade-off between force and displacement. A parametric simulation matrix was developed combining advanced materials—referencing commercial technical specifications [34,35]—and two actuator types (stack and bending) to examine mechanical outputs under varying voltage loads. These simulation findings are validated through experimental results to provide a framework for integrating simulation with experimental requirements.

The primary contribution of this work is the development of an integrated parametric simulation framework tailored specifically for a Polytetrafluoroethylene (PTFE) compliant mini-gripper, evaluating dynamic transient behavior and electromechanical efficiency. The remainder of this paper is structured as follows: Section 2 details the classification and properties of piezoelectric materials. Section 3 presents the analytical modeling and experimental case studies for both stack and bending piezoelectric actuators. Finally, Section 4 summarizes the conclusions and implications of the findings. This paper presents analytical and experimental results regarding the implementation of unconventional piezoelectric actuators within a compliant mechanism, specifically a mini gripper.

## 2. Materials and Methods

The development of high-performance compliant mechatronic systems fundamentally depends on the synergistic integration of two distinct material types: an active smart material that generates the electromechanical actuation and a passive structural material that elastically transmits and amplifies the induced motion. The choice of these materials determines the available force, maximum displacement stroke, and dynamic frequency response. This study’s experimental framework centers only on Lead Zirconate Titanate (PZT) as the active actuation material and Polytetrafluoroethylene (PTFE) as the passive compliant structure.

### 2.1. Lead Zirconate Titanate (PZT) as the Active Actuation Material

Among active smart materials, piezoelectric ceramics, particularly Lead Zirconate Titanate (PZT), are the benchmark for precision micro-positioning due to their superior electromechanical coupling coefficients, high intrinsic stiffness, and quick response times. The actuation mechanism in PZT depends on the realignment of its internal ferroelectric domains when subjected to a high-voltage electric field. This domain-switching phenomenon creates significant mechanical strain and is the principal cause of the localized electromechanical hysteresis observed during dynamic operation.

A key characteristic of the PZT material utilized in this study is its structural anisotropy, enabling the ceramic to be utilized in different operational modes depending on the actuator’s geometric configuration. The stack actuator architecture utilizes the longitudinal piezoelectric effect (d33 mode), wherein the produced strain aligns with the applied electric field, resulting in exceptionally strong blocking forces. In contrast, the bi-morph bending design employs the transverse piezoelectric effect (d31 mode) to substantially enhance the displacement stroke. The intrinsic electromechanical adaptability of PZT renders it an optimal choice for assessing the force–displacement trade-offs in compliant devices.

### 2.2. Polytetrafluoroethylene (PTFE) as a Compliant Structural Material

While active actuation is driven by PZT elements, the structural integrity and motion transmission of the mechatronic system rely entirely on the passive material of the compliant framework. In this study, Polytetrafluoroethylene (PTFE) was selected to manufacture the monolithic compliant mini-gripper. PTFE is a highly versatile fluoropolymer that exhibits distinct mechanical characteristics critical for compliant mechanisms. Most notably, its relatively low Young’s modulus (E ≈ 400 N/mm^2^) and Poisson’s ratio (ν = 0.39) provide the necessary high intrinsic compliance, allowing the rigid piezoelectric stroke to be elastically converted into a larger clamping motion without exceeding the material’s yield strength.

Furthermore, PTFE offers exceptional fatigue resistance, which is paramount for flexure hinges that undergo continuous cyclic loading during micro-manipulation tasks. From an application perspective, PTFE’s high chemical inertness, biocompatibility, and extremely low coefficient of friction make it an optimal candidate for handling sensitive biological samples in biomedical micro-robotics, avoiding contamination or adhesion issues typical of metallic grippers.

### 2.3. Experimental Methods and Measurement Setup

To empirically validate the theoretical electromechanical framework and accurately capture the behavior of these materials under dynamic loads, a highly precise experimental test bench was developed. The dynamic displacement characteristics of both the standalone PZT actuators and the integrated PTFE gripper arms were measured using a high-resolution, non-contact optical laser displacement sensor (Keyence LK-G300 series, made by Keyence Ltd., Milton Keynes, UK paired with a UGG32 controller from the same company).

Dynamic control and data acquisition were facilitated by a central control unit (Analog Discovery 2, made by Digilent co NI, Austin, TX, USA), which generated precise low-voltage waveforms. These command signals were subsequently amplified by a high-voltage power amplifier (30× AMP), made by Alca Systems, Magurele, Romania to provide the 0–150 V driving range required to optimally excite the PZT elements. This synchronized, closed-loop measurement setup allowed for the simultaneous monitoring of the applied high-voltage signals and the resulting physical deformation, ensuring a rigorous empirical validation of the theoretical models presented in Section 3.

## 3. Piezoelectric Actuators: Case Studies

This case study consists of two complementary parts focused on piezoelectric actuation technologies. The first part investigates a stack-type piezoelectric actuator capable of transmitting very high forces with excellent precision and stiffness. Such actuators are widely used in applications requiring high load capacity and nanometer-scale positioning accuracy. The study analyzes its force transmission characteristics, structural integration, and performance under constrained motion conditions. The second part examines a bending-type piezoelectric actuator designed to achieve large displacements compared to stack actuators. This type of actuator is suitable for applications where extended motion range and flexibility are required. The study evaluates its displacement amplification, mechanical behavior, and potential integration into compliant mechanisms. Together, the two parts highlight the trade-off between force and large displacement in piezoelectric actuation systems. The combined analysis provides insight into selecting the appropriate actuator type depending on the functional requirements of precision engineering applications.

### 3.1. Analysis and Testing for Stack Piezoelectric Actuator

At first, the *stack* piezoelectric actuator was *analyzed* with its axial deformation capability and then everything is incorporated into the model of the compliant frame. The analysis of the stack piezoelectric actuator with following specifications was realized: maximum voltage of 150 V; maximum stroke, $\Delta L_{free}=\;18µm$; capacitance of 1350 nF; blocking force, $F_{b}=1000\;N$; resonant frequency of 65 KHz; Young’s modulus of 4.4 × 10^10^ N/m^2^ and overall dimensions of 6.5 mm × 6.5 mm × 18 mm [36].

#### 3.1.1. Fundamental Piezoelectric Constitutive Equations

To establish a rigorous scientific foundation for the macro-mechanical behavior of both stack and bending configurations, the system must be governed by the standard linear coupled constitutive relations of piezoelectricity [37]. These equations couple the mechanical variables—strain (S_i_) and stress (T_j_)—with the electrical variables—electric field (E_k_) and electric displacement (D_l_).

(1) $$S_{i}={\;s}_{\mathrm{ij}}^{E}+d_{\mathrm{ki}}E_{k}$$

(2) $$D_{l}={{\;d}_{\mathrm{lj}}T_{j\;+\;}\varepsilon }_{\mathrm{lk}}^{T}$$

where $s_{\mathrm{ij}}^{E}$ is the elastic compliance measured at a constant electric field, $d_{\mathrm{ki}}$ and $d_{\mathrm{lj}}$ represent the piezoelectric charge constants (electromechanical coupling coefficients), and $\varepsilon_{\mathrm{lk}}^{T}$ is the dielectric permittivity tensor at constant mechanical stress T.

For the stack piezoelectric actuator analyzed in Section 3.1, operation relies primarily on the longitudinal piezoelectric effect (d_33_ mode), where the mechanical strain and the applied electric field are collinear along the polarization axis (axis 3) [4]. Assuming a multi-layer stack containing n active ceramic layers, each of individual thickness t, the free longitudinal stroke (ΔL_free_) under zero external stress (T_3_ = 0) is derived from Equation (1) as

(3) $$\Delta L_{\mathrm{free}}=n\;\cdot \;d_{33}\cdot V$$

where V is the applied control voltage. Consequently, the macroscopic actuator stiffness (k_piezostack_) depends strictly on its geometric parameters—overall cross-sectional area A and length L_0_—and the material’s short-circuit Young’s modulus (Y^E^):

(4) $$k_{\mathrm{piezostack}\;}=\;\frac{A\;\cdot \;Y^{E}}{L_{0}}$$

By substituting Equation (4) into the macroscopic equilibrium framework, the blocking force (F_b_) generated when external displacement is fully constrained (ΔL = 0) maps directly back to the intrinsic material parameters:

(5) $$F_{b}=\;k_{\mathrm{piezostack}}\cdot \Delta L_{\mathrm{free}}\;=\;\frac{A\;\cdot \;Y^{E}\cdot n\;\cdot \;d_{33}\cdot V}{L_{0}}$$

#### 3.1.2. Analysis for Stack Piezoelectric Actuator

Before testing the characteristics of the real system, a preliminary analytical calculation is performed for the actuator without considering specific operating conditions such as environmental effects, vibrations, humidity, and other external influences.

The analytical equations for stroke under axial load of piezoelectric actuator are:

(6) $$\Delta L\;=\;\Delta L_{\mathrm{free}}-\frac{F}{k_{\mathrm{piezostack}}}$$

where ΔL_0_—free displacement of the stack piezoelectric actuator.

F—axial load; k_piezostack_—rigidity of the stack actuator.

But axial force F:

(7) $$F\;=\;k_{\mathrm{ext}}\cdot \Delta L$$

Stroke when it has an external load (3) can be calculated:

(8) $$\Delta L\;=\;\Delta L_{\mathrm{free}}-\frac{k_{\mathrm{ext}}\Delta L}{k_{\mathrm{piezostack}}}$$

Solve:

(9) $$\Delta L\;=\;\Delta L_{\mathrm{free}}\cdot \frac{k_{\mathrm{piezostack}}}{k_{\mathrm{piezostack}}+k_{\mathrm{ext}}}$$

And the generated force:

(10) $$F\;={\;k}_{\mathrm{ext}}\Delta L\;=\;\Delta L_{\mathrm{free}}\cdot \frac{k_{\mathrm{piezostack}}}{k_{\mathrm{piezostack}}+k_{\mathrm{ext}}}$$

Bloking force (F_b_) is when ΔL=0

(11) $$0=\Delta L_{\mathrm{free}}-\frac{F_{b}}{k_{\mathrm{piezostack}}}$$

(12) $$F_{b}={\;k}_{\mathrm{piezostack}}\cdot \Delta L_{\mathrm{free}}$$

where

(13) $$k_{\mathrm{piezostack}}=\frac{F_{b}}{\Delta L_{\mathrm{free}}}=\frac{1000}{18}=55.55\;{\times \;10}^{3}\;N/\mathrm{mm}$$

The displacement for piezoelectric stack actuator can be calculated for various values of upload forces with Equation (6).

**Figure 1:**
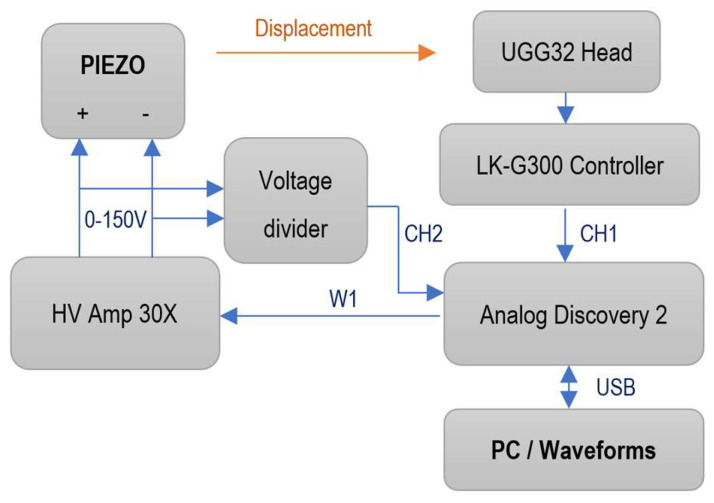
Command scheme for the piezoelectric actuator.

The command scheme for the piezoelectric actuator you can see in Figure 1. The system is designed to drive a piezo element with high voltage while simultaneously measuring its physical displacement using a laser displacement sensor. This schematic outlines a closed-loop or characterization setup for a piezoelectric actuator, using an Analog Discovery 2 (AD2) as the central controller and data acquisition unit.

The Drive Section (Control Path) generates the signal required to move the piezoelectric actuator. The Waveform Generator (Analog Discovery 2—W1) sends a low-voltage control signal (typically 0–5 V) to the high-voltage power amplifier (30X AMP). It takes the small signal from the AD2 and scales it up by a factor of 30. The actuator receives the amplified voltage (0–150 V). The input voltage (*V_in_*) leads to mechanical strain or movement in the piezo stack.

The Measurement Section (Feedback Path) tracks how much the piezo moves; a high-precision laser system is used. There is a laser displacement sensor head controller (UGG32), and a process optical signal (LK-G300). Differential Input (CH1+/CH1−): The analog output from the laser controller is fed into Channel 1 of the Analog Discovery 2. Using both positive and negative terminals suggests a differential measurement to reduce noise.

Data Acquisition and Monitoring CH2 (Monitoring): The output of the 30x Amplifier is looped back into Channel 2 of the AD2. This allows the user to monitor the actual high-voltage signal being applied to the piezo to ensure it matches the command signal. PC/Waveforms: The AD2 is connected via USB to a computer running Digilent Waveforms software, version 3.18.1. This is where the user generates the waveforms, views the oscilloscope traces (displacement vs. voltage), and records data.

#### 3.1.3. Testing the Stack Piezoelectric Actuator

**Figure 2:**
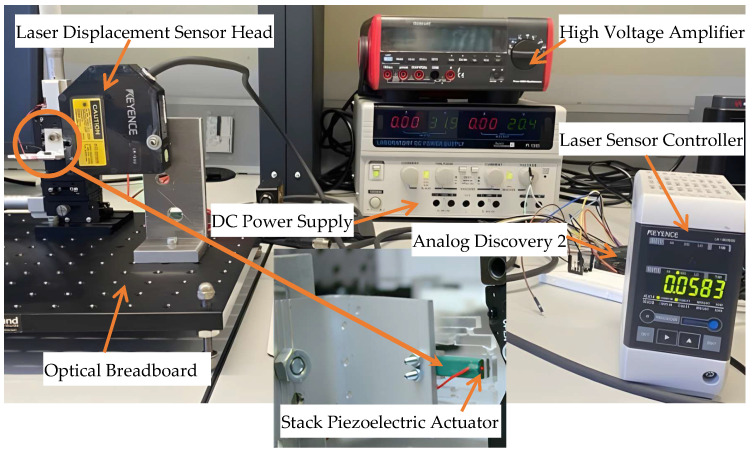
Testing bench for stack piezoelectric actuator.

Initially, the stack piezoelectric actuator is tested independently (Figure 2) using a high-precision piezoelectric characterization test bench used to measure the displacement of a piezo actuator relative to an input voltage (0 ÷ 150 V).

The PC (off camera) sends a waveform command to the AD2. The 30x AMP (powered by the DC supply, made by Multicomp Pro, Leeds, UK) boosts that signal to move the Piezo. The Laser Head detects the movement, and the Keyence Controller displays the value and sends a voltage back to the AD2.

**Figure 3:**
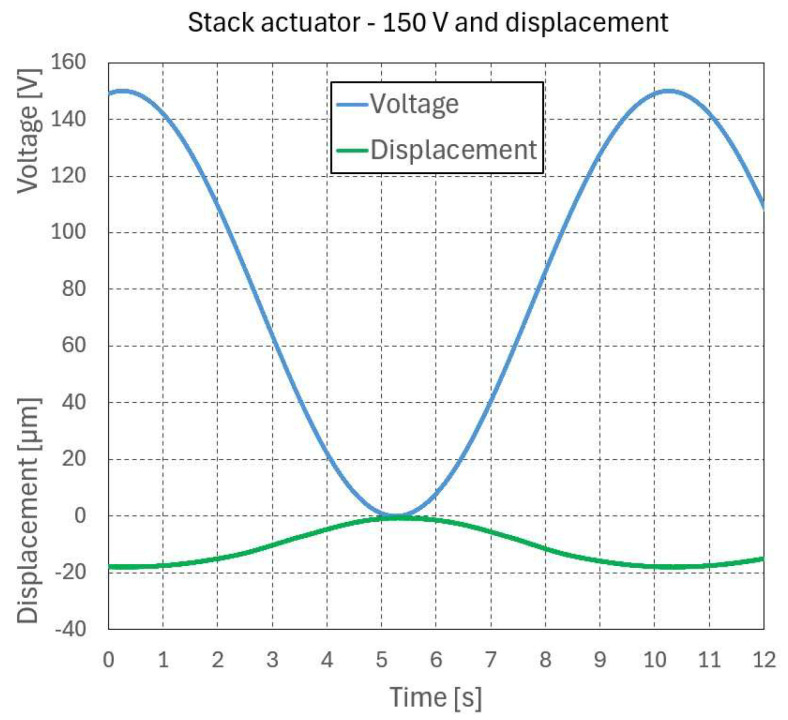
Testing results for stack piezoelectric actuator.

The user views a “Voltage vs. Displacement” curve on their screen to analyze the piezo’s performance (Figure 3).

#### 3.1.4. Testing the Stack Piezoelectric Actuator with a Compliant Gripper

**Figure 4:**
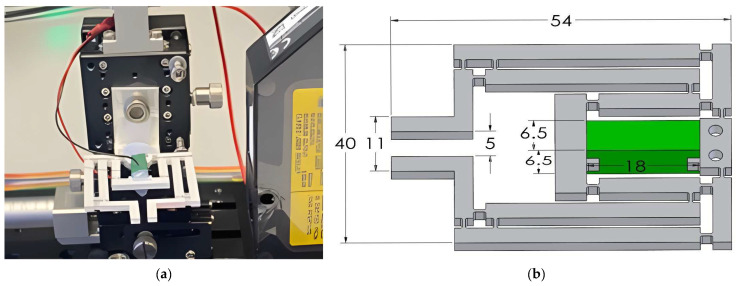
Testing bench (**a**) and 3D Model (**b**) for mini gripper with stack piezo actuator.

The next step was to evaluate the performance in a real system where the piezoelectric stack was implemented in a compliant gripper, made of Polytetrafluoroethylene PTFE (Young’s Modulus E = 400 N/mm^2^, and Poisson’s Ratio ν = 0.39) with overall dimensions 54 mm × 40 mm × 2 mm. The output displacement at the piezoelectric actuator will be input displacement for the gripper. The test bench for the mini-gripper (Figure 4a) is the same as the actuator test bench.

**Figure 5:**
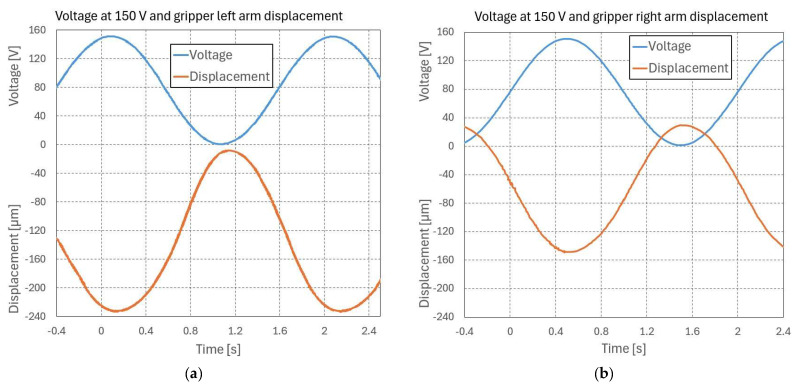
Mini gripper displacement for left arm (**a**) and right arm (**b**).

The graphical result from the Waveforms oscilloscope software shows the dynamic relationship between the drive voltage and the resulting physical displacement mini gripper of the left arm (Figure 5a) and right arm (Figure 5b).

The displacement curve (orange color) is roughly 180° out of phase with the voltage curve (blue color). When the voltage increases, the displacement signal reaches its minimum. This means the laser is measuring the distance to a surface that moves away or closer in inverse proportion to the applied voltage. Both signals appear to be clean sinusoids without significant clipping or distortion, suggesting the 30X AMP is operating within its linear range.

The low-voltage monitoring signal from the amplifier, oscillating between 0 V and 5 V. Scaled by the 30x amplifier, the drive voltage oscillates between 0 V and 150 V.

### 3.2. Analysis and Testing for Bending Piezoelectric Actuator

The *bending* piezoelectric actuator was analyzed with its axial deformation capability, and then everything was incorporated into the model of the compliant mini-gripper. The analysis of the bending piezoelectric actuator with following specifications was realized: maximum voltage 150 V; maximum stroke $\delta_{free}=\;450\;µm$; blocking force $F_{b}=1.5\;N$; resonant frequency: 370 Hz and overall dimensions are 32 mm × 7.8 mm × 0.8 mm [36].

#### 3.2.1. Analytical Foundation for the Bending Configuration

Unlike the longitudinal stack configuration, the bending piezoelectric actuator operates on the transverse piezoelectric effect (d_31_ mode) [38]. Here, the applied electric field along the polarization thickness axis (axis 3) induces a transverse strain along the longitudinal axis of the cantilever beam (axis 1). Under the simplified Euler–Bernoulli beam theory combined with Equation (1), the free tip displacement (ΔL_free_) for a bimorph bending actuator of length L, width w, and total thickness h is mathematically defined as

(14) $$\Delta L_{\mathrm{free}}=\frac{3}{2}\cdot \frac{L^{2}}{h^{2}}\cdot d_{31}\cdot V$$

The mechanical flexural stiffness of this cantilever configuration (k_piezobending_) is inherently lower than that of a solid stack due to the geometric moment of inertia and is expressed as

(15) $$k_{\mathrm{piezobending}}=\frac{w\;\cdot \;h^{3}\cdot Y^{E}}{4L^{3}}$$

This lower flexural stiffness explains the fundamental trade-off highlighted in this study: the bending configuration drastically amplifies free displacement at the expense of a diminished blocking force (F_b_), which is given by

(16) $$F_{b}=k_{\mathrm{piezobending}}\cdot {\Delta L}_{\mathrm{free}}=\frac{3}{8}\cdot \frac{w\;\cdot \;h^{3}\cdot Y^{E}\cdot d_{31}\cdot V}{L}$$

These structural physics relations dictate the macroscopic parameters used in the following equations and provide the scientific rationale for the performance discrepancies observed in our experimental testing.

#### 3.2.2. Analysis for Bending Piezoelectric Actuator

Before testing the characteristics of the real system, a preliminary analytical calculation is performed for the actuator without considering specific operating conditions such as environmental effects, vibrations, humidity, and other external influences.

The analytical equations for stroke under external load of piezoelectric actuator are [37]:

(17) $$\Delta L\;=\;{\Delta L}_{\mathrm{free}}-\frac{F}{k_{\mathrm{piezobending}}}$$

where ΔL_free_—free displacement of the bending piezoelectric actuator; F—axial load; k_piezobending_—rigidity of the bending actuator.

Stroke when it has an external load (3) can be calculated as follows:

(18) $$\Delta L\;=\;{\Delta L}_{\mathrm{free}}-\frac{k_{\mathrm{ext}}\Delta L}{k_{\mathrm{piezobending}}}$$

Solve:

(19) $$\Delta L\;=\;{\Delta L}_{\mathrm{free}}\cdot \frac{k_{\mathrm{piezobending}}}{k_{\mathrm{piezobending}}+k_{\mathrm{ext}}}$$

And the generated force:

(20) $$F\;={\;k}_{\mathrm{ext}}\Delta L\;=\;{\Delta L}_{\mathrm{free}}\cdot \frac{k_{\mathrm{piezobending}}}{k_{\mathrm{piezobending}}+k_{\mathrm{ext}}}$$

Bloking force (F_b_) is when δ=0

(21) $$F_{b}={\;k}_{\mathrm{piezobending}}\cdot {\Delta L}_{\mathrm{free}}$$

where

(22) $$k_{\mathrm{piezobending}}=\frac{F_{b}}{{\Delta L}_{\mathrm{free}}}=\frac{1.5}{450}\;=3.33\;N/\mathrm{mm}$$

The displacement for piezoelectric bending actuator can be calculated for various values of upload forces with Equation (17).

#### 3.2.3. Testing the Bending Piezoelectric Actuator

**Figure 6:**
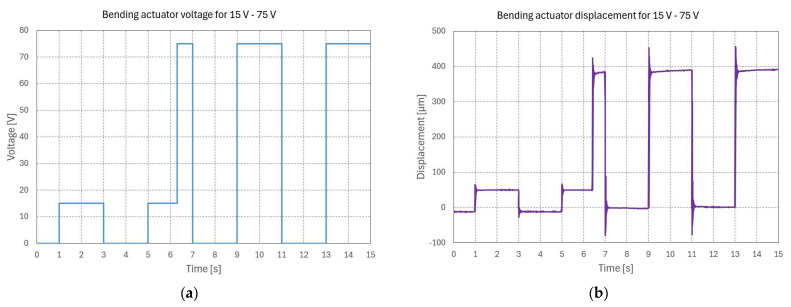
Piezobending voltage (**a**) and displacement (**b**).

Initially, the bending piezoelectric actuator is tested independently using the same test bench. The result of the hysteresis (Figure 6a) and the individual values in the figure are presented (Figure 6b). These two graphs show the performance of a bending piezoelectric actuator by comparing the electrical input (voltage) to the physical output (displacement) over a 15 s interval.

The control signal being sent to the actuator uses a series of square-wave pulses to test the actuator at Low Voltage 15 V and High Voltage 75 V (Figure 6a). The actuator shows a non-linear but consistent relationship between voltage and displacement (Figure 6b). However, it is underdamped, meaning it vibrates quite a bit before holding a steady position, especially at higher voltages. This graph measures how far the actuator stroke is as a response to the voltage: 50 µm for 15 V and 390 µm for 75 V. The actuator does not move perfectly to the target position. Every time the voltage jumps (on/off), there is a sharp spike or “overshoot” followed by rapid oscillations (ringing) before it settles. The overshoot is much more pronounced at 75 V, spiking to over 450 µm before settling back to 390 µm. For an input voltage 15 V it was a low-level movement and a minor overshoot.

#### 3.2.4. Dynamic Electromechanical Modeling and Transient Response Analysis

To rigorously analyze the physical mechanisms causing the underdamped oscillations and the significant overshoot observed at higher operating voltages (specifically at 75 V, as shown in Figure 6b), the compliant mini-gripper system driven by the bending piezoelectric actuator is modeled as a lumped-parameter second-order dynamic system. The electromechanical coupling, factoring in the constitutive piezoelectric relations and the structural damping of the Polytetrafluoroethylene (PTFE) compliant frame, is governed by the following differential equation:

(23) $$m_{\mathrm{eff}}\frac{d^{2}x(t)}{dt^{2}}\;+\;c\frac{\mathrm{dx}(t)}{\mathrm{dt}}\;+\;k_{\mathrm{eff}}x(t)\;=\;T_{\mathrm{em}}V\left(t\right)-F_{\mathrm{ext}}\left(t\right)$$

where x(t) represents the tip displacement of the actuator; m_eff_ is the effective moving mass; c is the equivalent viscous damping coefficient; k_eff_ is the total effective stiffness; T_em_ is the electromechanical transmission coefficient; V(t) is the applied step voltage excitation; and F_ext_(t) is the external resisting force.

By applying the Laplace transform under zero initial conditions, the voltage-to-displacement transfer function G(s) is expressed in the standard second-order form [39]:

(24) $$G(s)\;=\;\frac{K_{\mathrm{dc}}}{\frac{1}{\omega_{n}^{2}}s^{2}+\frac{2\zeta }{\omega_{n}}s+1}$$

where K_dc_ is the static DC gain, ω_n_ is the undamped natural frequency, and ζ is the dimensionless damping ratio. The experimental transient response illustrated in Figure 6b exhibits a classic underdamped behavior (ζ < 1). The maximum percentage overshoot (M_p_) recorded during the step response at 75 V is quantified using the experimental peak displacement (x_max_ = 450 μm) and the steady-state displacement (x_ss_ = 390 μm):

(25) $$M_{p}\;=\;\frac{x_{\max}-x_{\mathrm{ss}}}{x_{\mathrm{ss}}}\;\times \;100\%$$

From the experimental curve in Figure 6b, the measured overshoot is approximately 15.4%. Utilizing the analytical relationship between the overshoot and the damping ratio, the following is obtained:

(26) $$M_{p}\;=\;\exp\left(\frac{-\zeta \pi }{\sqrt{1\;-\;\zeta^{2}}}\right)$$

The structural damping ratio ζ of our system is mathematically estimated to be low (ζ ≈ 0.5). The severe ringing (vibrations) and high overshoot are mathematically attributed to this low damping ratio. This phenomenon occurs because the rapid charge accumulation on the internal capacitance of the PZT material induces an instantaneous electrostatic force that overcomes the high internal compliance of the PTFE framework before viscous dissipation takes effect. Furthermore, the localized electromechanical hysteresis acts as an unmodeled non-linear energy storage mechanism, aggravating the dynamic instability during the rising edge of the pulse. To mitigate this ringing in practical micro-positioning applications, a closed-loop control architecture is required.

### 3.3. Results

After analysis and testing, we obtained a relationship between voltage (input) and displacement (output) for three different displacements: stack piezo actuator, compliant mini-gripper and bending piezoelectric actuator configurations. It highlights how the displacement varies depending on the setup.

**Figure 7:**
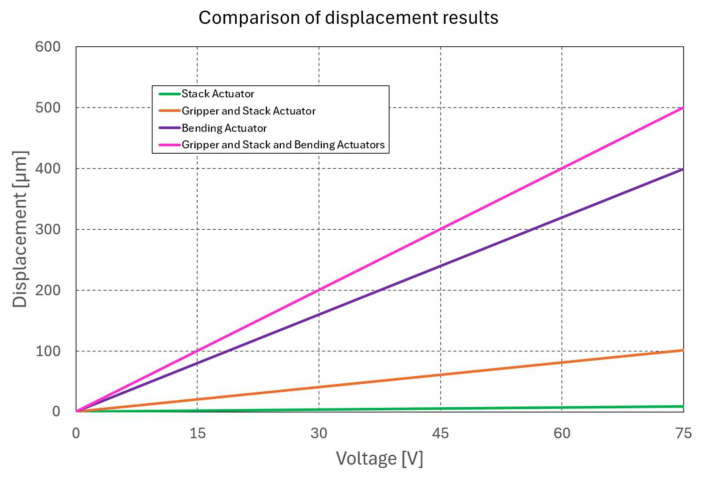
Displacements of actuators and grippers.

Figure 7 shows a comparative analysis between input voltage and displacement in three different configurations: the standalone stack actuator, the integrated compliant mini-gripper, and the bending piezoelectric actuator. The data shows that all three systems have a linear response to applied voltage. However, the piezoelectric bending design, shown by the purple curve, amplifies displacement more than the other two systems. The stack actuator keeps a minimum stroke, but the bending unit is significantly better at turning electrical energy into displacement, with values up to 450 µm. So, Figure 7 is an important design pattern that gives the empirical evidence needed to figure out the trade-offs between high-force, low-stroke precision (stack-based) and large-scale, flexible actuation (bending-based) for micro-scale systems. This graph finally proves that parametric modeling can be combined with experimental demands. It also gives a clear direction for choosing the best actuator based on the unique stroke-to-voltage efficiency needs.

## 4. Conclusions

This paper presents an analysis and experimental validation of the performance of piezoelectric actuators in compliant mechatronic systems. Experimental evidence demonstrated that the response of these actuators maintains a consistent linear correlation between input voltage and resulting displacement across various configurations. The comparative analysis indicates a significant performance settlement: stack piezoelectric actuators demonstrate superior force transmission and nanometer-level precision, whereas bending-type actuators have a considerably enhanced displacement range, achieving strokes of up to 450 µm.

The experimental results indicate that integrating these actuators into compliant mechanisms, such as the mini gripper developed in this study, offers a viable background for micro-scale manipulation and biological applications. However, the investigation also uncovered dynamic limitations in the operation of the bending actuator. Here are significant oscillations at high voltage levels (e.g., 75 V) compromising precision in the manipulation of highly sensitive biological cells. So, in this case it is necessary to improve the control of the piezo bending actuator. Specifically, to address the issue of high oscillations at high voltage levels, future implementations must integrate advanced closed-loop control strategies, such as active damping or decoupling compensators, to suppress vibrations and ensure precise manipulation.

In conclusion, this work successfully established an integrated parametric simulation framework tailored specifically for a PTFE compliant mini-gripper, rigorously evaluating both its dynamic transient behavior and electromechanical efficiency.

Future research directions will focus on the implementation of advanced closed-loop control algorithms to suppress the underdamped oscillations observed in bending actuators. Additionally, we plan to experimentally investigate customized piezoelectric composite materials to further optimize the trade-off between force and flexibility, alongside conducting a comprehensive cost–benefit analysis for scaling these compliant mechanisms in industrial applications.

## Data Availability

The original contributions presented in this study are included in the article. Further inquiries can be directed to the corresponding author.

## Abbreviations

The following abbreviations are used in this manuscript:

MEMS	Micro-Electro-Mechanical Systems
EAP	Electroactive Polymers
SMA	Shape Memory Alloys
FPI	Force-Dependent Prandtl–Ishlinskii (models)
PZT	Lead Zirconate Titanate
3D/4D	Three-Dimensional/Four-Dimensional (printing)
PTFE	Polytetrafluoroethylene
